# Correction: Mizuochi et al. Patients with Severe Trauma Having an Injury Severity Score of 24 and above Develop Nutritional Disorders. *Diagnostics* 2024, *14*, 1307

**DOI:** 10.3390/diagnostics15172182

**Published:** 2025-08-28

**Authors:** Minori Mizuochi, Junko Yamaguchi, Nobutaka Chiba, Kosaku Kinoshita

**Affiliations:** Division of Emergency and Critical Care Medicine, Department of Acute Medicine, Nihon University School of Medicine, Tokyo 173-8610, Japan; orita.minori@nihon-u.ac.jp (M.M.); chiba.nobutaka@nihon-u.ac.jp (N.C.); kinoshita.kosaku@nihon-u.ac.jp (K.K.)

In the original publication [1], there was a mistake in Table 2 as published. There were errors in the number of patients for each injury site of Table 2. The *p*-value of the Mann–Whitney U test also needs to be revised. The corrected Table 2 appears below.

The authors state that the scientific conclusions are unaffected. This correction was approved by the Academic Editor. The original publication has also been updated.

## Figures and Tables

**Table 2:** Characteristics of the patients on day 7 (*n* = 49).

	High CONUT Score Group (*n* = 24)	Low CONUT Score Group (*n* = 25)	*p*-Value *
Age (years)	60.0 (43.0–77.8)	49.0 (28.5–64.5)	0.023
Sex (M/F)	18:6	22:3	0.289
BMI	22.2 (20.6–26.7)	24.4 (20.0–25.5)	0.741
Site of injury			
Head	12	9	0.393
Spine	5	11	0.128
Chest	5	5	1.000
Abdomen	3	2	0.667
Extremities	12	9	0.393
Number of surgeries [*n*/ALL (%)]	18 [36.7]	11 [22.4]	0.042
Energy intake during 7 days (kcal)	5572.0 (3928.0–6808.5)	6266.0 (4977.0–9339.4)	0.072
APACHE II score	12.0 (7.0–15.0)	5.0 (2.0–9.0)	<0.001
SOFA score	3.0 (2.0–4.0)	0.5 (0.0–2.75)	0.007
ISS	25.0 (11.5–33.0)	16.0 (9.0–20.0)	0.022
Transthyretin (mg/dL)	28.3 (24.2–30.2)	26.0 (23.8–29.4)	0.529
Phosphorus (mg/dL)	3.2 (2.6–4.5)	3.1 (2.6–3.4)	0.332
Magnesium (mg/dL)	2.0 (1.8–2.4)	1.9 (1.8–2.1)	0.739
Zinc (μg/dL)	67.5 (54.0–79.5)	73.0 (62.0–79.0)	0.638
CONUT score at admission	2.0 (1.0–3.0)	1.0 (0.0–1.5)	0.008
Normal category	9	19	0.010
Light category	15	6	0.010
Lymphocyte count (/μL)	1581.1 (1000.7–2430.9)	2157.4 (1451.0–2780.6)	0.129
T-cho (mg/dL)	182.0 (151.8–205.3)	198.0 (162.5–214.0)	0.412
Albumin (g/dL)	4.1 (3.7–4.3)	4.3 (4.0–4.7)	0.023

* Continuous variables were compared using Student’s *t*-test or the Mann–Whitney U test, as appropriate. Chi-square or Fisher’s exact probability tests were performed for categorical variables. We determined the optimal cutoff points and significance level to be 5%. Abbreviations: M, male; F, female; APACHE II, Acute Physiology and Chronic Health Evaluation II; SOFA, Sequential Organ Failure Assessment; ISS, Injury Severity Score; CONUT score, Controlling Nutritional Status score; BMI, body mass index. High CONUT score group: CONUT score ≥ 5. Low CONUT score group: CONUT score ≤ 4.
