# Supplementary material for: Patients with Severe Trauma Having an Injury Severity Score of 24 and above Develop Nutritional Disorders
Source: Diagnostics (Basel). 2024 Jun 20;14(12):1307. doi: 10.3390/diagnostics14121307 (PMC11202517; doi:10.3390/diagnostics14121307)
Supplement: Supplementary file 1 [file diagnostics-14-01307-s001.zip › diagnostics-3021796-supplementary/FigS1, FigS2a,b 240608JY.pptx]

## Slide 1
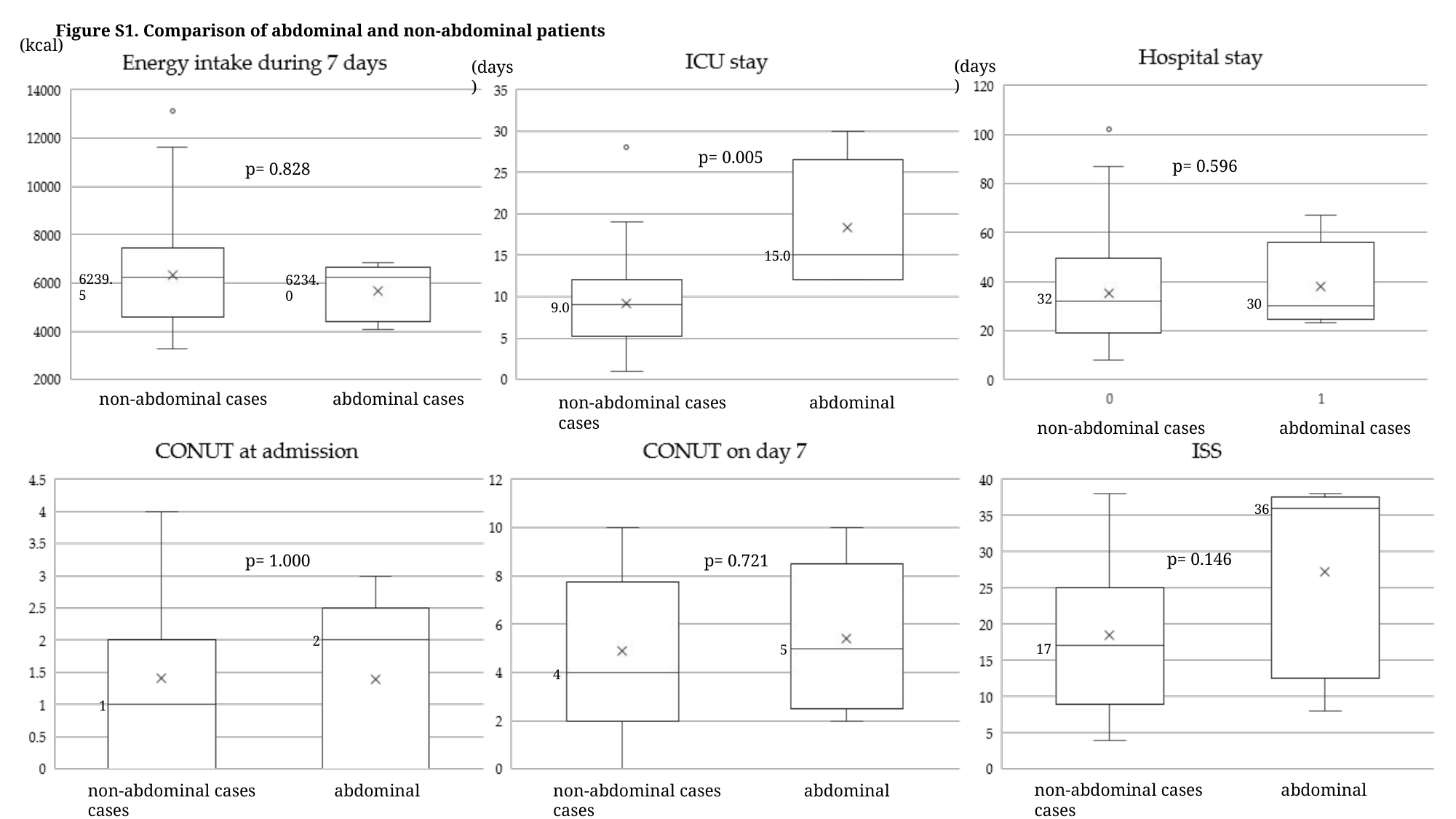

Figure S1. Comparison of abdominal and non-abdominal patients
(kcal)
(days)
(days)
p= 0.005
p= 0.596
p= 0.828
15.0
6239.5
6234.0
32
30
9.0
non-abdominal cases abdominal cases
non-abdominal cases abdominal cases
non-abdominal cases abdominal cases
36
p= 0.146
p= 1.000
p= 0.721
2
17
5
4
1
non-abdominal cases abdominal cases
non-abdominal cases abdominal cases
non-abdominal cases abdominal cases

## Slide 2
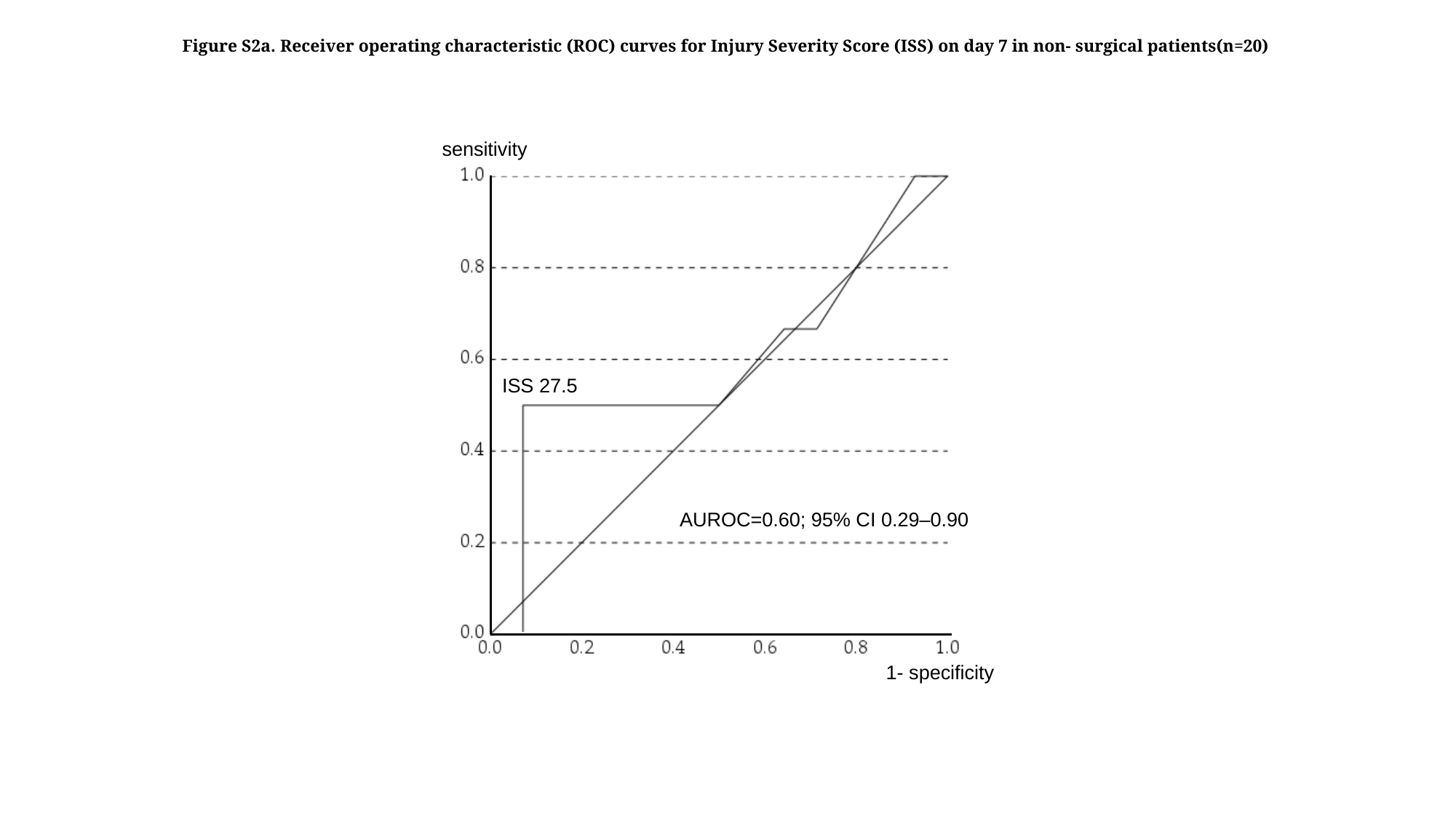

Figure S2a. Receiver operating characteristic (ROC) curves for Injury Severity Score (ISS) on day 7 in non- surgical patients(n=20)
sensitivity
ISS 27.5
AUROC=0.60; 95% CI 0.29–0.90
1- specificity

## Slide 3
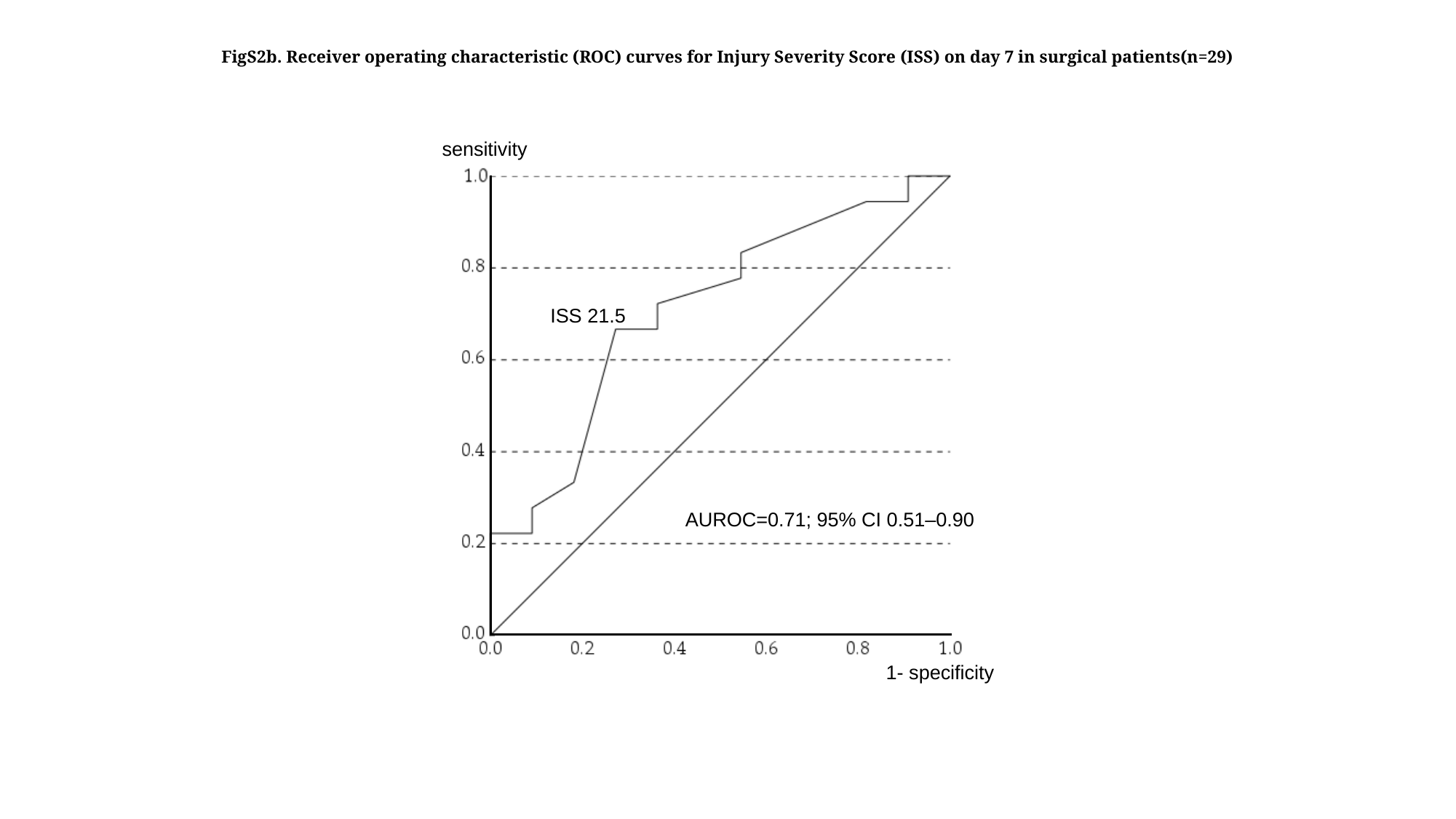

FigS2b. Receiver operating characteristic (ROC) curves for Injury Severity Score (ISS) on day 7 in surgical patients(n=29)
sensitivity
ISS 21.5
AUROC=0.71; 95% CI 0.51–0.90
1- specificity
