# Supplementary material for: Patients with Severe Trauma Having an Injury Severity Score of 24 and above Develop Nutritional Disorders
Source: Diagnostics (Basel). 2024 Jun 20;14(12):1307. doi: 10.3390/diagnostics14121307 (PMC11202517; doi:10.3390/diagnostics14121307)
Supplement: Supplementary file 1 [file diagnostics-14-01307-s001.zip › diagnostics-3021796-supplementary/Table S1 Trauma site 240608JY.pdf]

Table S1. Characteristics by trauma site

	Head(n=21)	Spine(n=16)	Chest(n=10)	Abdomen(n=5)	Extremities(n=21)
Energy intake during 7 days（kcal）	6298(4000-6506)	5778(4238-8734)	6318(4463-6956)	6234(4410-6645)	6298(3801-7476)
Sufficiency rate* (%)	45.7(37.8-55.3)	51.5(39.8-72.0)	50.1(34.0-66.6)	57.8(42.4-67.6)	56.0(35.1-69.0)
ISS	25.0(17.0-27.0)	17.0(16.0-28.8)	30.0(20.8-36.5)	36.0(12.5-37.5)	16.0(9.0-34.5)
CONUT at admission	1.0(0.0-3.0)	2.0(0.3-3.0)	2.0(0.8-3.3)	2.0(0.0-2.5)	1.0(0.0-2.0)
CONUT on day 7	6.0(3.0-8.0)	3.0(1.0-5.0)	4.5(3.8-7.0)	5.0(2.5-8.5)	5.0(3.0-7.0)
ICU stay (days)	10.0(7.0-13.5)	9.5(6.5-13.5)	12.0(9.5-14.5)	15.0(12.0-26.5)	8.0(4.0-14.5)
Hospital stay (days)	42(14.5-58.0)	31.0(24.0-41.3)	32.5(13.0-52.6)	30.0(24.5-56.0)	26.0(19.5-46.0)

Abbreviations: *ISS*, Injury Severity Score; *CONUT*, Controlling Nutritional Status; *ICU*, Intensive Care Unit.

*Sufficiency rate=Energy intake during 7 days/ 25kcal×standard body weight×7 days
