# Supplementary material for: Patients with Severe Trauma Having an Injury Severity Score of 24 and above Develop Nutritional Disorders
Source: Diagnostics (Basel). 2024 Jun 20;14(12):1307. doi: 10.3390/diagnostics14121307 (PMC11202517; doi:10.3390/diagnostics14121307)
Supplement: Supplementary file 1 [file diagnostics-14-01307-s001.zip › diagnostics-3021796-supplementary/Table S2 Surgcal and Non-Surgical 240608JY.pdf]

Table S2. Comparison of severity and energy intake between non-surgical treatment and surgical treatment

	Non-surgical treatment	Surgical treatment	p-value*
ISS	16.5(9.0-24.3)	25.0(9.0-26.0)	0.525
CONUT at admission	1.0(0.3-2.0)	1.0(0.0-2.5)	0.698
CONUT on day 7	3.0(1.3-5.8)	6.0(4.0-8.5)	0.004
Energy intake during 7 dayss (kcal)	6422(5295-7450)	5550(3948-6494)	0.029
Sufficiency rate (%)**	53.8(46.4-68.3)	44.0(33.4-63.1)	0.206

* Continuous variables were compared using Student's t-test or the Mann–Whitney U test, as appropriate.

Chi-square tests were performed for categorical variables.

**Sufficiency rate=Energy intake during 7 days/ 25kcal×standard body weight×7 days

We determined the significance level to be 5%.

Abbreviations: *ISS*, Injury Severity Score; *CONUT*, Controlling Nutritional Status.
